# Outcome after Reconstruction of the Proximal Tibia – Complications and Competing Risk Analysis

**DOI:** 10.1371/journal.pone.0135736

**Published:** 2015-08-13

**Authors:** Stephan E. Puchner, Paul Kutscha-Lissberg, Alexandra Kaider, Joannis Panotopoulos, Rudolf Puchner, Christoph Böhler, Gerhard Hobusch, Reinhard Windhager, Philipp T. Funovics

**Affiliations:** 1 Department of Orthopedics, Vienna General Hospital, Medical University of Vienna, Waehringer Guertel, Vienna, Austria; 2 Center for Medical Statistics, Informatics, and Intelligent Systems—Section for Clinical Biometrics, Medical University of Vienna, Waehringer Guertel, Vienna, Austria; 3 Rheumatologist in private practice, Wels, Austria; VIT University, INDIA

## Abstract

**Background and Objectives:**

The proximal tibia (pT) is a common site for bone tumors. Improvements in imaging, chemotherapy and surgical technique made limb salvage surgery the treatment of choice. Yet, reconstructions of the pT have been associated with less favorable outcome compared to other parts of the extremities. The aim of this study was to evaluate the outcome of patients with a modular endoprosthetic reconstruction of the pT.

**Methods:**

Eighty-one consecutive patients with an average age of 29 years underwent endoprosthetic reconstruction of the pT. Postoperative complications were categorized according to the ISOLS classification, and revision-free survival until first complication (any Type 1–5), soft tissue failure (Type 1), aseptic loosening (Type 2), structural failure (Type 3), infection (Type 4), and local tumor progression (Type 5) was estimated by using a Fine-Gray model for competing risk analyses for univariate and multivariable regression with Firth’s bias correction.

**Results:**

A total of 45 patients (56%) had at least one complication. Cumulative incidence for complication Types 1 to 5 at 5 years with death and amputation as competing events revealed a risk of 41% for the first complication, 14% for Type 1, 16% for Type 2, 11% for Type 3, 17% for Type 4, and 1% for Type 5.

**Conclusion:**

Despite inclusion of amputation and death as strong competing events, pT replacements are still associated with a high risk of postoperative failures. The results suggest that infection and soft tissue failures (Type 1 and 5) seem to depend from each other. Sufficient soft tissue reconstruction and closure allow better function and reduce the risk of infection as the most prominent complication. The use of a rotating hinge design has significantly reduced structural failures over time.

## Introduction

The proximal tibia (pT) counts as the second most common site for primary bone tumors [1, 2]. Tumors of the pT includes up to 15% of all osteosarcomas, 11% of Ewing sarcomas and 6% of chondrosarcomas [2, 3]. Until the late 1970s, the standard treatment for tumors around the pT was amputation above the knee [3]. Today, after dramatic changes in the management of these lesions over the past decades and advances in imaging, surgical techniques and prosthetic designs, limb salvage surgery became the standard treatment method for tumors located at the pT [1, 4–6]. There are several techniques for limb salvage in the pT including endoprosthetic reconstruction [1, 3, 7], osteoarticular allograft or allograft prosthesis composite [6, 8], resection and arthrosthesis [9] and rotationplasty [10, 11]. However, due to its anatomical environment, tumor resection and especially endoprosthetic reconstruction of the proximal tibia faces certain problems. The immediate proximity to the peroneus nerve, as well as to the popliteal vessels go along with a difficult surgical approach followed by poor options for soft tissue coverage. In addition, in most cases reconstruction of the extensor mechanism is necessary and several different types of reconstructions have been described [4–6, 12]. Consequently pT reconstructions are associated with a higher number of complications compared to other limb salvage procedures, ranging between 40 to 70% at 5-15yeras [1, 13, 14]. These include infections, structural failures, aseptic loosening, local recurrence and a high number of soft tissue failures [3, 7, 14–23]. To further standardize the graduation of prosthesis related complications Henderson et al. [24] proposed a classification in 2011 and adopted it in 2014 to become accepted by the International Society of Limb Salvage (ISOLS classification) [25]. Soft tissue failure was considered as Type 1, aseptic loosening as Type 2, component breakage and periprosthetic fracture as Type 3, infection as Type 4, and local recurrence as Type 5.

The aim of this study was to investigate surgical outcomes and, in particular primary and subsequent complications of endoprosthetic reconstruction of the pT after resection of musculoskeletal tumors using a competing risk model.

## Material and Methods

A retrospective cohort study was performed using prospectively collected data from our local bone and soft tissue tumor registry between 1983 and 2009. Seventeen patients (21%) underwent operation before 1990, 35 patients (43%) between 1990 and 1999, 29 patients (29%) after 2000. Limb salvage surgery for a tumor around the pT, using a modular endoprosthetic replacement, was performed in 87 patients. Six (7%) patients were excluded from the study because of inadequate historical data. All other patients have been included in this retrospective single-center study. Approval of the institutional review board was obtained prior to this investigation (Ethical Review Board—Medical University of Vienna—EK Nr: 767/2008). All patient records/information was anonymized and de-identified prior to analysis. All medical records and patient files of our database have been reviewed. Table 1 gives a detailed description of demographic and treatment variables of all 81 included patients.

**Table 1:** Baseline characteristics and operative data.

Variable	Number
**Patients**	81
**Sex**	
Males	49 (60%)
Females	32(40%)
**Age at time of surgery (Years; SD)**	29±16
**Median follow-up (Months; Range)**	139 (1–359)
Osteosarcoma	44 (54%)
Pleomorphic undifferentied sarcoma	6 (7%)
Ewing’s Sarcoma	4 (5%)
Chondrosarcoma	4 (5%)
Others	10 (12%)
Metastatic disease	8 (10%)
Giant cell tumor	5 (6%)
**Staging (MSTS/Enneking)**	
Primary malignant	68 (83%)
1a	2 (3%)
1b	1 (2%)
2a	2 (3%)
2b	60 (74%)
3a	3 (4%)
**Type of prosthesis**	
KMFTR (first/second generation, fixed hinge)	59 (73%)
GMRS (third generation, rotating hinge)	22 (27%)
Cemented	4 (5%)
**Mean length of resection (mm)**	150±40 (70–250)
**Soft tissue coverage**	
Gastrocnemius muscle flap	58 (72%)
Latissimus dorsi free muscle flap	5 (6%)
Split skin graft	36 (44%)
**Extensor mechanism reconstruction**	
Fibula transposition	24 (30%)
Synthetic ligament	18 (22%)
Gastrocnemius +PDS	23 (28%)
**Adjuvant Therapy**	
Chemotherapy	59 (73%)
Radiotherapy	16 (20%)
Chemotherapy + Radiotherapy	10 (8%)

The median follow-up of all patients was 139 months (range, 1–359 months). Fourteen patients (17%) had a follow-up of less than two years; ten of these have died of their underlying disease, the remaining four patients had no complication within the first two post-operative years. There were 49 males (60%) and 32 females (40%) with an average age of 29±16 years (range, 11 to 77 years) at the time of index surgery. Fifty-two (64%) patients were under 30 years of age at time of surgery.

The diagnosis was always based on conclusive clinical and imaging findings (conventional radiography, computed tomography (CT), magnet resonance imaging (MRI) of the primary site and in more recent cases arteriograms, scintigrams and PET-CT) and was always confirmed by biopsy and histological analysis by experienced pathologists specialized in orthopedic oncology at our institution.

Sixty-eight (83%) had a primary malignant bone tumor (six of these patients (7%) had distant metastases at the time of diagnosis). Eight patients (10%) had metastatic disease. The primary cancer in patients with metastases was renal cell carcinoma (two patients), mamma carcinoma (one patient), squamous cell carcinoma (one patient), prostate cancer (one patient), plasmocytoma (two patients), and undifferentiated (one patient). Five patients (7%) had a malignant giant cell tumor.

The decision regarding the treatment of our patients was always made in a multidisciplinary tumor board, involving surgeons, oncologists, radiologists, radiotherapists and pathologists. Surgery was always performed according to the principals of musculoskeletal tumor surgery.

The Kotz Modular Femur and Tibia Reconstruction System (first generation); (KMFTR, Howmedica GmbH, Kiel, Germany) or the Howmedica Modular Reconstruction System (second generation); (HMRS, Howmedica GmbH, Kiel, Germany) was implanted in 59 patients (73%) since 1983. More recently, since 2003, the Global Modular Replacement System (third generation); (GMRS, Stryker Corp., Mahwah, NJ) was implanted in 22 patients (27%). The main difference of the latter prosthetic design consists of a cementless stem fixation including a hydroxyapatite coating and titanium spray, as well as a rotating hinge design. A cemented stem fixation with a gentamycin loaded bone cement (Palacos R+G; Heraeus Medical, Hanau, Germany) was performed in four patients (5%). All other 77 patients had a cementless press-fit fixation of the stem. The mean length of the prosthesis was 150±40 mm (range, 70 to 250 mm). In 58 patients (72%) an additional soft tissue reconstruction with a gastrocnemius muscle flap was performed for coverage of the endoprosthsis. Another five patients (6%) required a latissimus dorsi free muscle flap. A split skin graft for soft tissue coverage was used in 36 patients (44%). Sixty-five patients (80%) had an extensor repair: Eighteen (22%) with a synthetic ligament (LARS). Transposition of the fibula in 24 patients (30%). Gastrocnemius + PDS (ETHICON Products · Robert-Koch-Straße 1 · D-22851 Norderstedt) in 23 patients (28%).

Patient mobilization was allowed from the first post-operative day. Initially, all patients had splints or braces for six weeks after surgery. Subsequently all patients were advised to wear braces for further six weeks by-weekly increases in range of flexion by 30° (0°-30°-60°-90°).

### Statistical analysis

Data analysis focused on the surgical outcome and complications of pT reconstruction. Primary endpoint of the study was a first revision of the pT prosthesis for any complication, secondary endpoints were complications classified as Type 1 to 5. Structural failure (Type 3) was regarded periprosthetic or prosthetic fracture or deficient osseous supporting structure or bushing wear. Investigated prognostic factors were age (under 30/over 30), sex, prosthetic design (rotating hinge/fixed hinge) and the use of a synthetic ligament. Descriptive summary statistics included means and frequencies. Differences between means and proportions were tested with the Chi-square test for categorical variables and the t-test for continuous variables. The median follow-up time was calculated using the inverse Kaplan-Meier method [26]. Overall survival probability was estimated using the Kaplan-Meier (KM) method. Cumulative incidence of complications was estimated in a competing risk (CR) model, where death and amputation of the affected extremity were modeled as competing events. Different types of complications were not defined as competing events, as patients suffering from a specific type of complication were still regarded at unchanged continuous risk to subsequently suffer from any other type of complication (e. g. infection after aseptic loosening). Also, in the analysis for a specific type, complications from other types were not censored. A separate CR analysis was performed for the first complication over time (irrespective of its type) and for each type of complication. The time interval to a respective endpoint (first complication and types 1 to 5) was defined as time from first implantation of the prosthesis to the time of the first occurrence of the complication type of interest, irrespective of the potential occurrence of other complications in between. A Fine-Gray model [27] for CR was used for univariate and multivariable regression analyses. Additionally, Firth’s bias correction was applied using the SAS macro PSHREG [28] to avoid overestimation of the effects due to the rather small number of events in the multivariable Fine-Gray models for first complication and complications 1 to 5. No analysis for cofactors was calculated for Type 5 complications due to too low number of events (N = 3). To test for possible differences in the incidence of complications over the period of treatment (defined as decades before the year 1990, between 1990 and 1999, and after the year 2000) this factor was additionally tested in separate univariate Fine-Gray models. All statistical tests were two-sided. A p-value of <0.05 was considered statistically significant. Calculations were performed using the software SAS (version 9.4; 2002–2012 by SAS Institute Inc., Cary, NC, USA).

## Results

Wide resection margins were achieved in 70 patients (86%), marginal margins in ten patients (12%) and one patient (1%) with metastatic disease underwent an intralesional resection. At latest follow-up, 51 patients (63%) had no evidence of disease (NED), nine patients (11%) were alive with disease (AWD), 20 patients (25%) died of disease (DOD). Fourteen of these died without suffering any complication before death. The overall disease specific survival of all patients according to KM was 91%, 79% and 78% at 1, 5 and 10 years, respectively. Secondary amputation was indicated in four patients (5%) due to local recurrence in two and recurrent infection in two.

### Overall prosthetic survival & Complications

**Fig 1:**
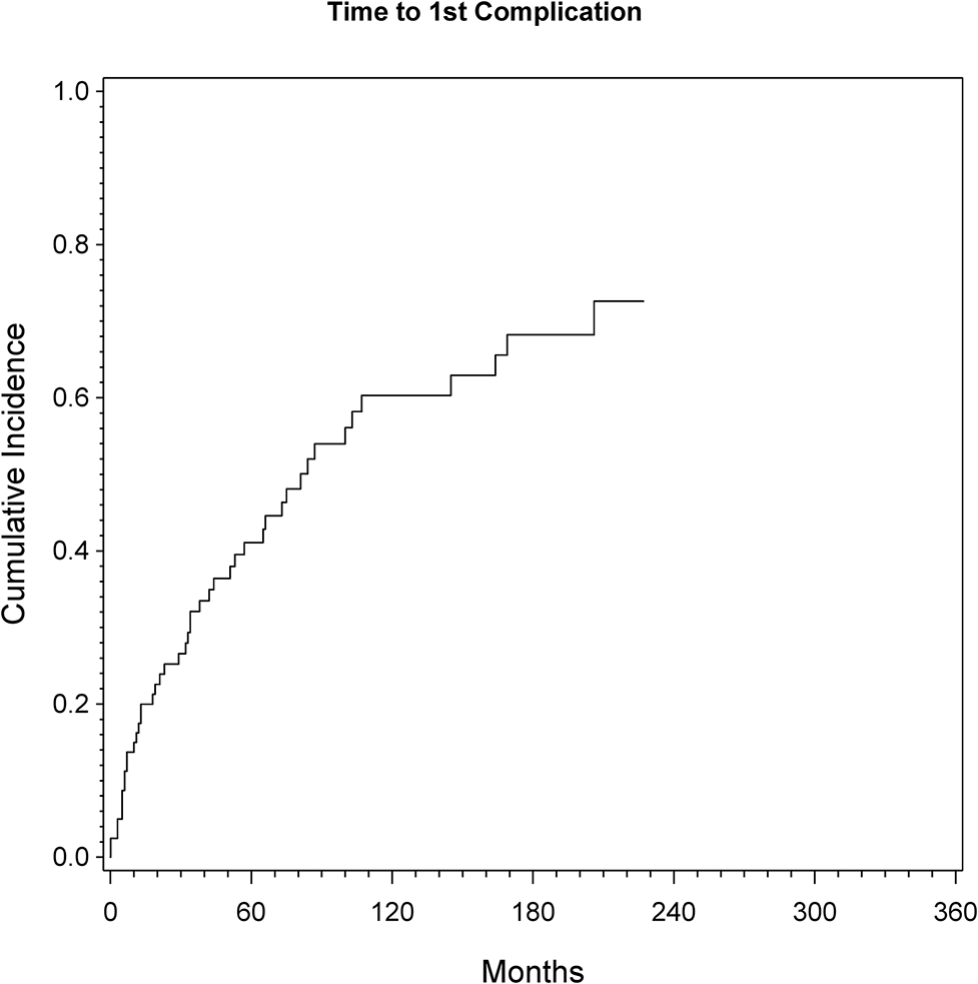
Cumulative incidence of first complication (any Type 1 to 5) as assessed by CR analysis (see material and methods for details).

A total of 45 patients (56%) had at least one complication according to the previous described classification. Estimation of cumulative incidence revealed an overall risk of first complication and revision of the modular prosthesis of the proximal tibia of 17% at one year, 41% at five years and 60% at ten years. None of the considered prognostic factors showed a statistically significant influence in univariate or multivariable analyses, yet there was a trend toward more complications for patients under the age of 30 years (univariate p = 0.067, HR = 1.79; multivariable p = 0.074, HR = 1.77; see Table 2 and Fig 1). The overall cumulative risk for any complication was not significantly dependent from the decade of treatment.

**Table 2:** Univariate and multivariable analyses of 1st and type 1 to 4 complications based on a Competing Risks analysis.*

		Univariate			Multivariable	
	HR	95%CI	p-value	HR	95%CI	p-value
**1st Complication**						
Sex (male/Female)	1	0,55–1,80	0,994	1,01	0,55–1,85	0,988
Age (<30/>30)	1,79	0,94–3,42	0,067	1,77	0,92–3,43	0,074
Prostetic design (rotating/fixed)	0,62	0,28–1,37	0,206	0,86	0,30–2,48	0,775
Synthetic ligament (yes/no)	0,63	0,27–1,466	0,254	0,7	0,23–2,13	0,531
**Type 1 complication**						
Sex (male/Female)	1,3	0,51–3,31	0,569	1,373	0,54–3,52	0,499
Age (<30/>30)	0,9	0,35–2,31	0,821	0,855	0,33–2,21	0,741
Prostetic design (rotating/fixed)	0,55	0,14–2,20	0,354	0,596	0,11–3,18	0,547
Synthetic ligament (yes/no)	0,68	0,17–2,67	0,546	0,926	0,17–4,99	0,931
**Type 2 complication**						
Sex (male/Female)	0,88	0,30–2,56	0,807	0,91	0,31–2,65	0,854
Age (<30/>30)	1,21	0,42–3,54	0,707	1,3	0,44–3,88	0,62
Prostetic design (rotating/fixed)	1,58	0,45–5,41	0,485	2,38	0,47–12,1	0,348
Synthetic ligament (yes/no)	1,16	0,28–4,75	0,831	0,64	0,09–4,16	0,649
**Type 3 complication**						
Sex (male/Female)	0,85	0,35–2,07	0,707	0,97	0,40–2,36	0,94
Age (<30/>30)	1,22	0,50–2,98	0,646	1,1	0,45–2,69	0,83
Prostetic design (rotating/fixed)	0,3	0,05–1,68	0,097	0,97	0,18–5,30	0,973
Synthetic ligament (yes/no)	0,13	0,01–1,95	*0,022	0,12	0,01–2,54	0,115
**Type 4 complication**						
Sex (male/Female)	1,56	0,64–3,78	0,321	1,55	0,63–3,79	0,32
Age (<30/>30)	0,81	0,33–1,99	0,63	0,77	0,31–1,91	0,56
Prostetic design (rotating/fixed)	0,92	0,36–3,13	0,923	0,73	0,16–3,25	0,682
Synthetic ligament (yes/no)	1,41	0,48–4,20	0,537	1,7	0,38–7,60	0,512

HR—hazard ratio; CI—confidence interval of HR

* Type 5 complications excluded for too low number of events

### Type 1 (soft-tissue failure)

**Fig 2:**
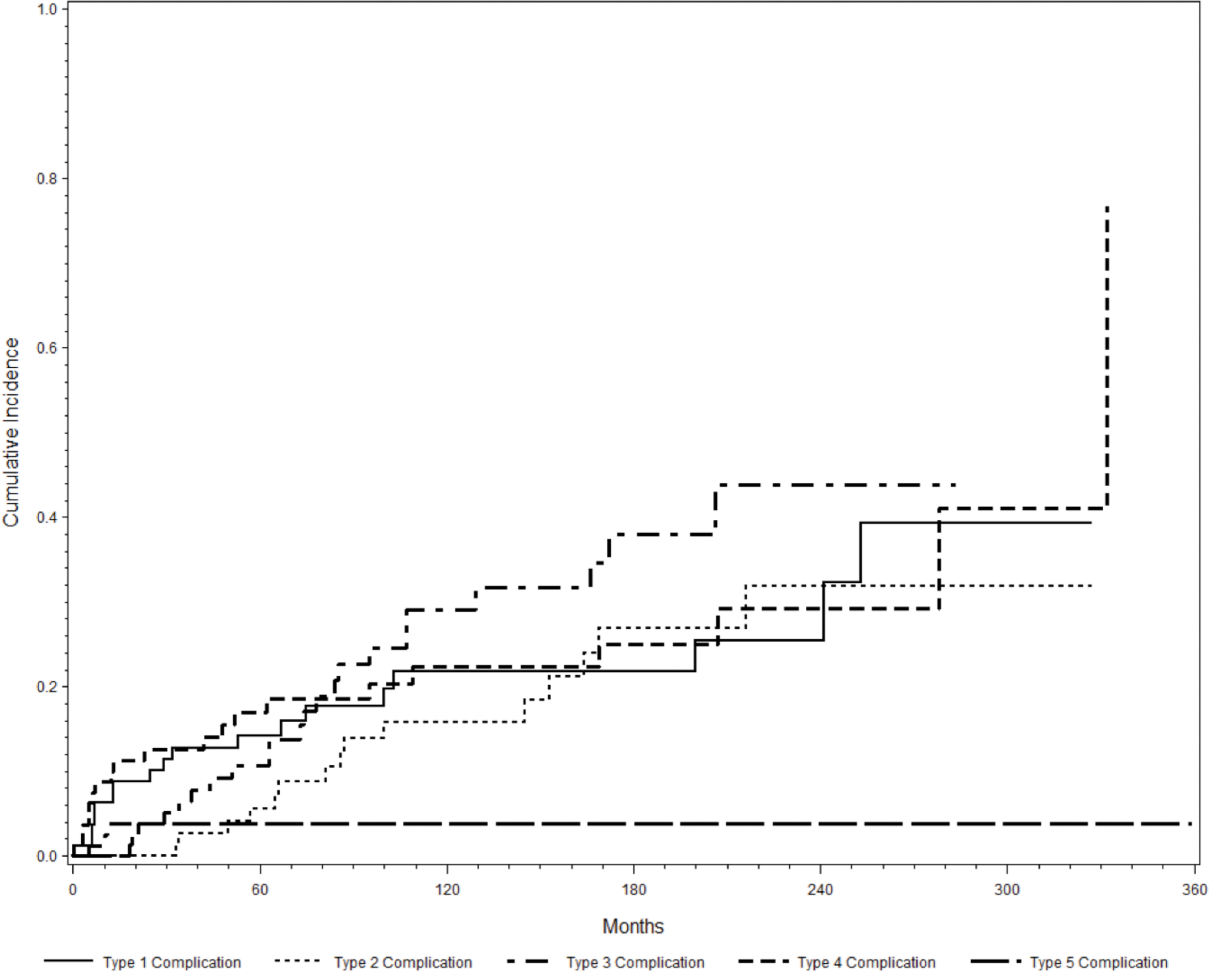
Cumulative incidence of complication types 1 to 5 over time as assessed by separate CR analysis for each type (see material and methods for details).

Eight patients (10%) experienced a Type 1 complication after index surgery and underwent revision (six for extensor mechanism insufficiency and two for wound dehiscence). In ten other patients (12%) Type 1 failure occurred as subsequent complication after revision for infection in four, aseptic loosening in two, structural failure in three and minor wound revision in one patient. Three patients who initially suffered from a Type 1 failure had to be revised again due to the same complication. Eight of 18 (44%) patients with a Type 1 failure also experienced a failure of Type 4, whereas only 12 out of 63 patients with no Type 1 failure experienced a Type IV failure. The risk for soft tissue failure was estimated as 6%, 14% and 21% at 1, 5, and 10 years, respectively. None of the considered prognostic factors showed a statistically significant influence on Type 1 complications in univariate or multivariable analysis (see Table 2 and Fig 2). There was no significant change in the incidence of soft tissue failures or the respective cumulative risk throughout the different decades of treatment.

### Type 2 (aseptic loosening)

Ten patients (12%) experienced aseptic loosening of the primary implant. One of these within two years after initial implantation of the prosthesis and two patients suffered from recurrent aseptic loosing after the prosthesis was initially exchanged for aseptic loosening. Five patients (6%) experienced aseptic loosening of the implant, which had earlier been revised due to a Type 3 complication. The estimated probability of experiencing aseptic loosening according to CR analysis was 6% and 16% at 5 and 10 years. None of the considered prognostic factors showed a statistically significant influence on Type 2 complications in univariate or multivariable analyses (see Table 2 and Fig 2). There was no change in the incidence of aseptic loosening or the respective cumulative risk throughout the different decades of treatment.

### Type 3 (structural failure; including bushing wear)

Twelve patients (15%) initially sustained a Type 3 complication before experiencing any other complication (bushing wear in seven and prosthetic fracture in five). Of these twelve patients, seven sustained a recurrent structural failure (bushing wear in six and prosthetic fracture in one). Ten patients experienced a Type 3 complication (bushing wear in six and periprosthetic fracture in four) after previously being revised for a Type 2 complication in four, a Type 1 complication in three, a Type 4 complication in two and a minor wound revision in one patient. Three of these had to be revised recurrently due to a structural failure. Overall 22 patients experienced a Type 3 complication. The competing risk model revealed an estimated risk for this type of complication of 4%, 11% and 29% at 1, 5, and 10 years, respectively. The use of a synthetic ligament was a statistically significant prognostic factor for a lower risk of a Type 3 failure according to the univariate CR analysis (p = 0.002), yet this could not be confirmed by multivariable analysis. All other prognostic factors were not statistically significant in univariate and multivariable analysis (see Table 2 and Fig 2). Structural failures were the only type of complications, which were statistically dependent from the decade of treatment (p = 0.04), with a continuously lower cumulative risk from the years 1990 to 1999 (HR = 0.46) and after 2000 (HR = 0.22) as compared to patients treated before 1990.

### Type 4 (infection)

Ten patients (12%) experienced an infection of their primary implant, which lead to one stage revision in nine patients and two-stage revision in one patient. Ten additional patients (12%) sustained an infection of the prosthesis following prior revision surgery due to other complications (four had structural failures, three had soft tissue failures, two had aseptic loosening and one patient had multiple revisions due to structural failure and aseptic loosening before developing a late infection 26 years after initial surgery. Eight of 20 (40%) patients with a Type 4 failure experienced also a failure of Type 1 failure, whereas only 10 out of 61 (16%) patients with no Type 4 failure experienced a Type 1 failure. CR analysis showed a cumulative incidence of infection of 7%, 17% and 22% at 1, 5, and 10 years, respectively. Neither of the tested prognostic factors revealed a statistically significant influence on developing an infection in univariate and multivariable analyses (see Table 2 and Fig 2). Again, there was no change in the incidence of infection or the respective cumulative risk throughout the different decades of treatment.

### Type 5 (tumor progression/local recurrence)

Three patients (4%) developed local recurrence after a mean time of 19 months after surgery (two with a malignant giant cell tumor and one with fribrosarcoma). Competing risk analysis revealed a risk for local-recurrence of 1% at five years and 4% at ten years. Two patients had to be treated by secondary amputation. The remaining patient with local recurrence of a malignant giant cell tumor was successfully treated by tumor resection. None of the prognostic factors was a statistically significant predictor in univariate analyses. No analysis of cofactors and no comparison of incidence over time were applied due to the small number of events.

#### MSTS

The mean functional score according to the Musculoskeletal Tumor Society was 82% (range, 37%- 100%). MSTS function was not significantly different between patients with or without complications, patients with fixed or rotating hinge prostheses, and patients with different extensor mechanism reconstructions.

## Discussion

Limb-salvage surgery for extremity sarcoma has increased over the last decades due to improved surgical techniques, higher prosthetic survival aroused through further developments in modular prosthetic design, and greater experience using these types of prostheses [1, 3]. In addition, reconstructions like osteoarticular allografts and allograft-prosthesis composites provide an alternative to endoprostetic reconstructions and allow restoration of bone stock, biological reattachment of host tendons, ligaments, and capsule. Most of these reconstructions are technically more demanding than endoprosthetic reconstructions and are associated with allograft-related complications such as non-union, fracture, subchondral collapse, articular cartilage degeneration, and instability [1, 20, 29–34]. Mankin et al found that 55% of patients require at least one reoperation after allograft reconstruction for bone tumors [35]. On the other hand prosthetic reconstructions allow rapid return to weight bearing and a shorter period of immobilization. Yet, modular endoprosthetic reconstructions of the proximal tibia have been associated with less favorable outcome compared to other parts of the extremities [5, 7, 13, 14, 36, 37]. High rates of complications have been reported after these procedures, which include aseptic loosening, structural failures, soft tissue failures and high rate of infections [23, 24]. So far, however, none of these reports incorporated a CR model with death and amputation as obvious and strong competing events in patients with (1) a malignant tumor and (2) a high rate of therapeutic failures.

To analyze our data, we therefore performed, to our best knowledge the first CR analysis focusing on pT endoprosthetic reconstructions. Earlier we have shown that CR analysis results in considerably lower risk estimates for all types of endoprosthetic complications in our series with osteosarcoma patients and that these lower failure rates may better reflect reality resulting from the high competing risk of death of disease and also amputation. As a consequence this might reflect a more realistic description of endoprosthetic survival in oncologic patients [38]. Yet further studies using this statistical analysis are needed to compare these results and draw conclusions, which go beyond a single center experience.

Overall, 45 patients (55%) suffered from one or more complications in this series and CR analysis revealed a risk of 17% at one year, 41% at two years and 60% at ten years to experience at least one complication, again showing the high number of complications after this procedure. The most common complication was structural failure (22 patients) followed by infection (20 patients).

We have to acknowledge several limitations of this study. First, this is a retrospective single center analysis. A follow-up of less than two years in some patients will still capture all deaths but will tend to de-emphasize oncologic failures relative to deaths. Nevertheless, accurately reviewed patient data in a retrospective study delivers useful results to obtain conclusion for management of further treatment. Inclusion of all patients treated in the study period irrespective of their actual length of follow-up is essential to obtain unbiased estimates of the complication incidences. Especially, patients dying early of their underlying disease should not be ignored despite short follow-up since they are typical for the disease under consideration and representative for future patients. Secondly, the group of indications is heterogeneous. Patients included in this study suffered from primary sarcoma as well as malignant aggressive giant cell tumors and bone metastases. Some patients were administered adjuvants, which may affect results. Some had a low life expectancy, while others had a high. In this context, it must be admitted that the interpretation of outcome may be affected by oncological outcomes and life expectancy, yet with CR analyses some of this bias will be reduced, especially for prosthetic survival. Given the fact that these factors will have a major impact on patients’ overall survival rates, we did not further analyze this data in detail. Since prospective randomized controlled trials are not available in this area of reconstruction and might not be in the future, our results can be useful, even under consideration of these limitations.

Reviewing literature, revision rates of proximal tibia reconstructions are varying between 40% and 73% at 5 years and 45 to 50% at 10 and 15 years [1, 3, 7, 13, 14, 16, 18, 19, 37]. Like other authors we found a high rate of complications and revisions in our series. Grimer et al reported, that by five years 40% of patients had a further surgery of some sort and by ten years even 70%. Gosheger et al found a 5-year prosthetic survival of 62% for proximal tibia reconstruction in his series about endoprosthetic reconstructions in sarcoma patients. It also has to be considered that a large portion of these patients are young and otherwise fit and healthy. These patients are very active and occasionally will not hesitate to put exceptional stress to their prosthesis, which will make revision surgery more common. Although it was not significant, we found a trend towards more complications for patients under the age of 30 years (univariate: p = 0.067, HR = 1.79; multivariable p = 0.074, HR = 1.77). In this regard substantial reconstruction of the proximal tibia with preservation of function is necessary.

Another major factor associated with clinical outcomes after proximal tibia replacement is function of the extensor mechanism after major soft-tissue resections. Many methods were described, including direct reattachment of the patellar tendon to the prosthesis [5], reinforcement with autologous bone-graft [39], and attachment of the patellar tendon to the transposed gastrocnemius muscle flap [5, 14, 36, 40], transposition of the fibula [5, 41] and synthetic ligaments [42, 43]. There is consensus about the need of direct attachment of the extensor mechanism to the prosthesis for adequate healing and scarring [1, 4, 37, 40, 42, 43]. We found a significant impact of synthetic ligament in terms of Type 3 complications in univariate analysis, yet could not strengthen these parameter by multivariable analysis. Otherwise, similar to Mavrogenis et al we could not find any significant association of different types of extensor reconstruction in terms of prosthesis survival and function [1].

Complications related to soft tissue reconstructions differ from 9% to 40% [1, 3, 5, 14, 36]. Our data analysis showed a Type 1 failure rate of 10% and 22% for the first revision and overall revisions. As may be expected most of them were extensor mechanism insufficiencies. In literature poor soft tissue coverage has been connected to higher rates of infections and wound healing problems [1, 3, 4, 13, 14, 36]. In this context we found that eight of 18 (44%) patients with a Type 1 failure experienced also a failure of Type 4.

Aseptic loosening has been reported one the most common failures throughout literature [24]. In our series this type of failure occurred in 11% after initial surgery and 18% overall. Unwin et al. reported that the probability of a patient surviving aseptic loosening for 10 years was 58% for a proximal tibia implant [13]. In our series it was 24% at 10 years. On the other hand Mavrogenis et al. presented a very low rate of aseptic loosening of 6% [1] in his recently published series.

Structural failure, such as fracture, insufficient bony support or bushing wear was the most common type of failure (24% to first, 36% overall) to revision in this study. Since some authors tend to exclude bushing wear in their data and different types of periprosthetic fractures are not included in these studies it is difficult to compare our data. Myers et al found, that 19% of patients in their series about proximal tibia reconstruction required at least one or more re-bushing [7]. Although it might be a technically simple procedure, it is still an open revision of the implant and naturally will introduce a higher risk of infection to the prosthesis. Interestingly, we have not observed a change of the cumulative risk of first complication or the different types of complications except type 3 between the different decades of treatment (before the year 1990, 1990 to 1999, and after 2000). Since the newer generation of prosthetic design with a rotating hinge (GMRS) became available in the year 2003, this fact may be influenced by the change in joint anatomy, reducing stress raisers on surrounding bone and the prosthetic material, although we found no statistical difference between fixed and rotating hinge prosthesis in our analysis.

Infection was the most common mode of failure in Henderson’s investigation (15,1%) in 2011. It was 11% after initial surgery and 23% over all in this series. A similar infection rate of 12% was reported by Lee et al [16]. Several authors reported to have objected a reduction of infection, when using a gastrocnemius muscle flap. Myers et al. reported an infection rate of 31% without a muscle flap and 14% with a flap in their 194 patients. Grimer et al. showed a decrease of their infection rates from 36% to 12%, by the use of a gastrocnemius muscle flap for soft tissue coverage [3]. Surprisingly neither of the tested prognostic factors revealed a significant influence on developing an infection in univariate and multivariable analyses in our series and we could find no association between infection rates and the use of a muscle flap. Even though flap coverage was not correlated to higher infection rates in their recently published series, Marvrogenis recommended the use of a gastrogenmius flap reconstruction of the extensor mechanism and for wound coverage, again underlying the important dependency of soft tissue coverage and infection [1].

Like in other studies local recurrence rates, especially for primary tumors and giant cell tumors were low, which shows that resections of the proximal tibia allow excellent tumor control. Myers et al found their risk of local recurrence declined from 10.6% do 4% after 1996 [7]. In this context reconstruction of the proximal tibia with a prosthesis is an oncologically adequate alternative to amputation.

## Conclusion

Complication rates after pT reconstruction are high, and literature shows a great variation of results. Despite inclusion of amputation and death as strong competing events, pT replacements are still associated with a high risk of postoperative failures. The results suggest that infection and soft tissue failures (Type 1 and 5) seem to depend from each other. Sufficient soft tissue reconstruction and closure allow better function and reduce the risk of infection as the most prominent complication. The use of a rotating hinge design has significantly reduced structural failures over time.
